# Milk BHB Heritability Estimates and Genetic Correlations with Milk Yield in Grazing Dairies in Chile

**DOI:** 10.3168/jdsc.2025-0938

**Published:** 2026-05-20

**Authors:** Guanxi Tao, Mingxuan Cai, Pedro Melendez

**Affiliations:** 1 Department of Biostatistics, City University of Hong Kong, Hong Kong SAR; 2 Department of Veterinary Clinical Sciences, Jockey Club College of Veterinary Medicine and Life Sciences, City University of Hong Kong, Hong Kong SAR

## Abstract

This study aimed to estimate the heritability of BHB in milk and evaluate its genetic correlations with other traits in grazing dairy herds from southern Chile. The analysis was based on a comprehensive dataset provided by a DHIA cooperative (Cooprinsem, Osorno, Chile). The dataset included records from 617 grazing farms and 712,629 lactations spanning from 2019 to 2024. The final dataset included 254,979 lactations from 50,794 cows. Milk BHB concentrations were higher in early lactation and remained relatively low and stable thereafter. Heritability estimates for BHB followed a parabolic trend, with the lowest values observed during early and late lactation and a pronounced peak of ~0.20 occurring in month 7. Genetic correlations between BHB and other traits were notably stronger than their phenotypic counterparts, suggesting robust underlying genetic relationships. In particular, the genetic correlation between BHB and milk yield shifted markedly over time, starting at approximately 0.35 ± 0.02 in month 1 and declining to approximately −0.30 ± 0.03 by month 10, whereas the phenotypic correlation remained weakly negative throughout the lactation period.

## Highlights

• Milk BHB concentrations in grazing cows are lower than in confined cows.
• Milk BHB heritability ranges from 0.12 to 0.20 across the entire lactation.
• Genetic correlation between milk BHB and milk yield was moderately positive to low toward mid lactation.

## Graphical Abstract

This study examined milk β-hydroxybutyrate (BHB) concentrations in Chilean grazing cattle from an original dataset of 617 farms and 712,629 lactations, evaluating a final dataset of 254,979 lactations from 50,794 cows from 2019 to 2024. Milk BHB concentrations remained stable throughout lactation (0.05 ± 0.04 mmol/L). Heritability estimates for BHB followed a parabolic trend, with the lowest values observed during early and late lactation, and a peak of approximately 0.20 in month 7. Genetic correlations between BHB and milk yield and fat and protein percentages were stronger than their phenotypic counterparts, suggesting robust underlying genetic relationships. HE = Haseman-Elston; MUN = milk urea nitrogen; SCS = somatic cell score.

**Figure:**
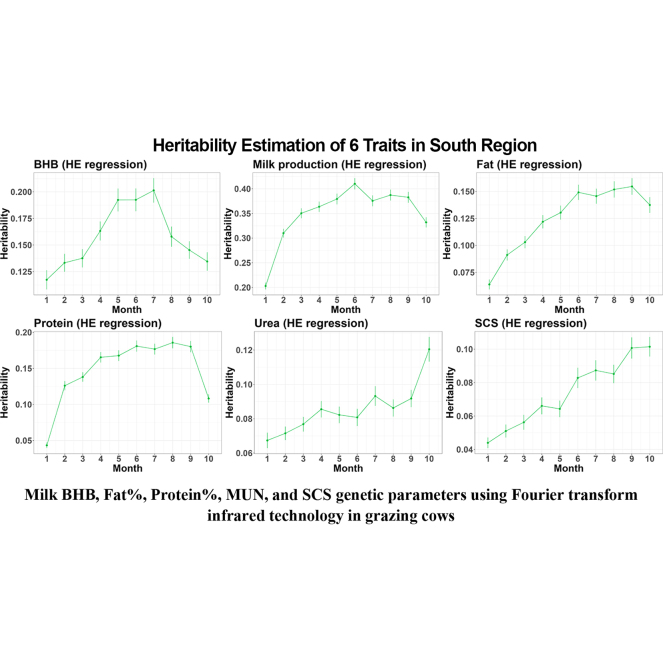

In postpartum dairy cows, high energy demands for milk production combined with reduced feed intake around parturition create a negative energy balance, leading to greater ketone body synthesis, primarily BHB (Melendez and Serrano, 2024). When BHB levels exceed physiological thresholds, subclinical ketosis may occur, especially within the first 7 DIM, adversely affecting lactational performance, fertility, and overall health (McArt et al., 2013). Blood BHB testing is costly and labor intensive (Sailer et al., 2018). Consequently, milk BHB measurement via mid-infrared spectroscopy during routine monthly testing by dairy herd improvement associations is a cost-effective alternative that has gained popularity in recent years. Milk BHB shows a strong correlation with blood BHB (r = 0.80), with sensitivity and specificity of 81% and 92%, respectively (de Roos et al., 2007; Renaud et al., 2019).

Heritability estimates for milk BHB and its genetic correlations with milk traits, such as fat or protein percentage, suggest the potential to develop health and metabolic selection indices. However, contemporary genetic evaluations of milk BHB and its association with other constituents in grazing cattle are not well explored. This study aimed to estimate milk BHB heritability and assess genetic correlations in grazing herds from southern Chile monitored by an official DHIA.

Chile has a dairy cattle population of ~350,000 head, with ~85% of cows located in the southern region managed in grazing-based systems (Fedeleche, 2025). The dairy sector in southern Chile is centered around coordinates 39.82°S, 73.23°W; 40.12°S, 72.38°W; 41.39°S, 73.46°W; and 41.43°S, 72.94°W. This region has cool average temperatures of 7°C in autumn and 10°C in spring. Rainfall is high, averaging 850 mm in autumn and winter and 150 mm in spring and summer (Chilean Weather Center, 2025). Approximately 90% of farms follow a biseasonal parturition, with 35% of cows calving in fall and 65% in spring, and cows are milked twice daily (Chilean Dairy Consortium, 2025). The forage base consists mainly of ryegrass pastures (*Lolium perenne*), with seasonal variation in usage depending on pasture growth rates. The cows are fed a variable amount of dry matter through grazing, depending on the season of the year and the farm management. The difference is provided as mixed rations before each milking, supplemented with silage such as oat, ryegrass, and corn. In addition, a variable amount of concentrate is offered in the milking parlor according to the cow's production level and DIM.

As part of routine herd management, ketone body concentrations, such as BHB in blood or milk or acetoacetate in urine, are typically monitored during the first 10 d postpartum. Cows exhibiting elevated levels, indicative of subclinical or clinical ketosis, are treated in accordance with established veterinary protocols.

This study consisted of the analysis of a comprehensive dataset from a DHIA cooperative (Cooprinsem, Osorno, Chile), from 617 grazing farms and 712,629 lactations from 2019 to 2024. The final dataset included 254,979 lactations from 50,794 cows. Cows could contribute multiple lactations over the study period, resulting in repeated records per animal. Frequency for each test day (**T1–T10**) was T1 = 11.42%, T2 = 11.28%, T3 = 11.09%, T4 = 10.89%, T5 = 10.65%, T6 = 10.37%, T7 = 10.02%, T8 = 9.51%, T9 = 8.46%, and T10 = 6.31%. Assessment of the daily milk yield of the cow was carried out by 2 official evaluators. A composite milk sample from each cow's quarter was obtained on each milking in sanitized plastic containers to assess fat (%), total protein (%), MUN (mg/dL), SCC (1,000/mL), and BHB (mmol/L). Samples were processed on a MilkoScan 7 RM (FOSS, Hillerød, Denmark). The dataset was downloaded from the machine software to a Microsoft Excel spreadsheet. Then the data were preprocessed by using the R software (4.1.2, R Core Team, 2022). First, records with missing sire or dam information were removed from the dataset. This was followed by the removal of erroneous records where a cow's ID was identical to its mother's ID. The dataset was further filtered to include only animals with available breed information and was subsequently filtered again to retain only records from the southern region. In addition, basic data cleaning was applied before estimation. We removed records with missing values for all the traits and retained only finite observations after any transformation. For example, SCC was transformed as SCS = 3 + log_2_(SCC/100) and then filtered for finite values. The cleaned data were then used to derive residuals from models including breed, year-by-dairy (left control), lactation, days dry, and seasons for downstream analyses. After these preprocessing steps, the final dataset used for the analysis consisted of 254,979 records from 50,794 cows. The pedigree-based relationship matrix (**GRM**) was then computed using the nadiv R package (Wolak, 2012). The GRM was constructed using the full available pedigree, including ancestors without phenotypic records, to avoid bias associated with pedigree trimming. The matrix was subsequently converted to the Genome-wide Complex Trait Analysis (GCTA) software GRM format and used as input in GCTA for variance component estimation. This approach ensures appropriate estimation of additive genetic variance and follows standard practice in animal breeding studies.

We estimated the variance and covariance components of milk BHB and other constituents with the mixed effects model. Instead of the REML approach that requires normality assumption on the phenotypes, we adopted the moment-based Haseman–Elston (**HE**) regression for parameter estimation. The reasons are 2-fold. First, the distribution of milk BHB concentrations is highly right-skewed and deviates substantially from normality. We have carefully evaluated the distributional properties of our milk BHB concentrations and explored log-transformation and inverse normal transformation strategies. However, due to the large proportion of near-zero values and measurement noise inherent to milk BHB obtained via mid-infrared spectroscopy, these transformations did not sufficiently normalize the distribution. Moreover, transformation complicates the biological interpretation of variance components and genetic parameters. Given our large sample size (>250,000 lactations), the HE regression provides consistent and statistically efficient estimates without requiring strict normality assumptions. Second, our analysis included 254,979 lactations from 50,794 cows, a dataset considerably larger than those used in most previous studies. At this scale, applying REML as implemented in standard software such as DMU (https://dmu.ghpc.au.dk/dmu/) becomes computationally substantial for repeated analyses across multiple lactation stages and traits. The use of a moment-based approach in this study substantially improves computational efficiency while retaining statistical consistency and efficiency in large samples. Therefore, we adopted the moment-based HE regression, which is robust to violations of normality assumptions and offers a computational advantage. We used the open-source tool GCTA (Yang et al., 2011) to fit the mixed effects model and obtained the parameter estimates and their SE. First, to estimate heritability, the linear mixed model can be formulated as follows:

**y** = **Xb** + **Zα** + **Wβ** + **e**,

where **y** is the phenotypic vector of a milk constituent, with each element representing a record of lactation; **X** is the matrix of covariates including herd coded as dummy variables, year of sampling, number of lactations, and days of dry period; and **Z** and **W** are the incidence matrices relating lactation records to individual cows, respectively. It was assumed that **b** is the vector of fixed effects for covariates, **α** is the vector of genetic effects with $\alpha ∼N\left(0,\sigma_{\alpha }^{2}A\right),$ $\beta ∼N\left(0,\sigma_{\beta }^{2}I\right)$ is the vector of the permanent environment effects, and $e\;\left(0,\sigma_{e}^{2}I\right),$ where **A** is the genetic relatedness matrix derived from the pedigree relationships. With the above specification, we can estimate the heritability of milk constituents as $h^{2}=\frac{\sigma_{\alpha }^{2}}{\sigma_{\beta }^{2}+\sigma_{\alpha }^{2}+\sigma_{e}^{2}}.$

To estimate genetic correlation between BHB and other milk constituents, we consider the following bivariate linear mixed model:

$$y_{1}=Xb_{1}+Z\alpha_{1}+W\beta_{1}+e_{1}$$

and

$$y_{2}=Xb_{2}+Z\alpha_{2}+W\beta_{2}+e_{2},$$

where **y**_1_ and **y**_2_ are phenotypic vectors for BHB and another milk trait, **b**_1_ and **b**_2_ are fixed effects of covariates, $\left(\begin{array}{c} \alpha_{1}\\ \alpha_{2} \end{array}\right)∼N\left(0,\Sigma_{\alpha }⊗A\right),$ $\left(\begin{array}{c} \beta_{1}\\ \beta_{2} \end{array}\right)∼N\left(0,\Sigma_{\beta }⊗I\right),$ $\Sigma_{\alpha }=\left(\begin{array}{cc} \sigma_{\alpha 1}^{2} & \sigma_{\alpha 12}^{2}\\ \sigma_{\alpha 12}^{2} & \sigma_{\alpha 2}^{2} \end{array}\right),$ $\Sigma_{\beta }=\left(\begin{array}{cc} \sigma_{\beta 1}^{2} & \sigma_{\beta 12}^{2}\\ \sigma_{\beta 12}^{2} & \sigma_{\beta 2}^{2} \end{array}\right),$ and ⊗ is the Kronecker product. Then, the genetic correlation for each month can be obtained as $r_{g}=\frac{\sigma_{\alpha 12}^{2}}{\sqrt{\sigma_{\alpha 1}^{2}\times \sigma_{\alpha 2}^{2}}}.$ The genetic correlations were estimated at each month within a lactation.

The average monthly values for the 6 evaluated traits showed distinct trends across the first 10 mo of lactation (Figure 1). Milk production followed a standard lactation curve, which peaked in the second month at 29.16 ± 9.22 kg and subsequently declined. In contrast, the trends for fat and protein percentage were largely inverse to that of milk production. Both traits reached their lowest values in the second month before steadily increasing through the later lactation stage. Milk BHB concentrations were elevated at T1 (0.06 ± 0.04 mmol/L) but stabilized thereafter, remaining within a narrow range of 0.03 to 0.04 mmol/L from T2 through T9. Milk urea nitrogen values rose to a peak in the seventh month. We also observed that the mean for SCS showed a continuous and marked increase from the second month onward, which was aligned with previous findings. Overall, these phenotypic trends illustrate a lactation period defined by 2 distinct phases. The first phase is characterized by a peak in milk production, followed by a second phase of rising fat and protein percentages and a progressive increase in SCS.

**Figure 1:**
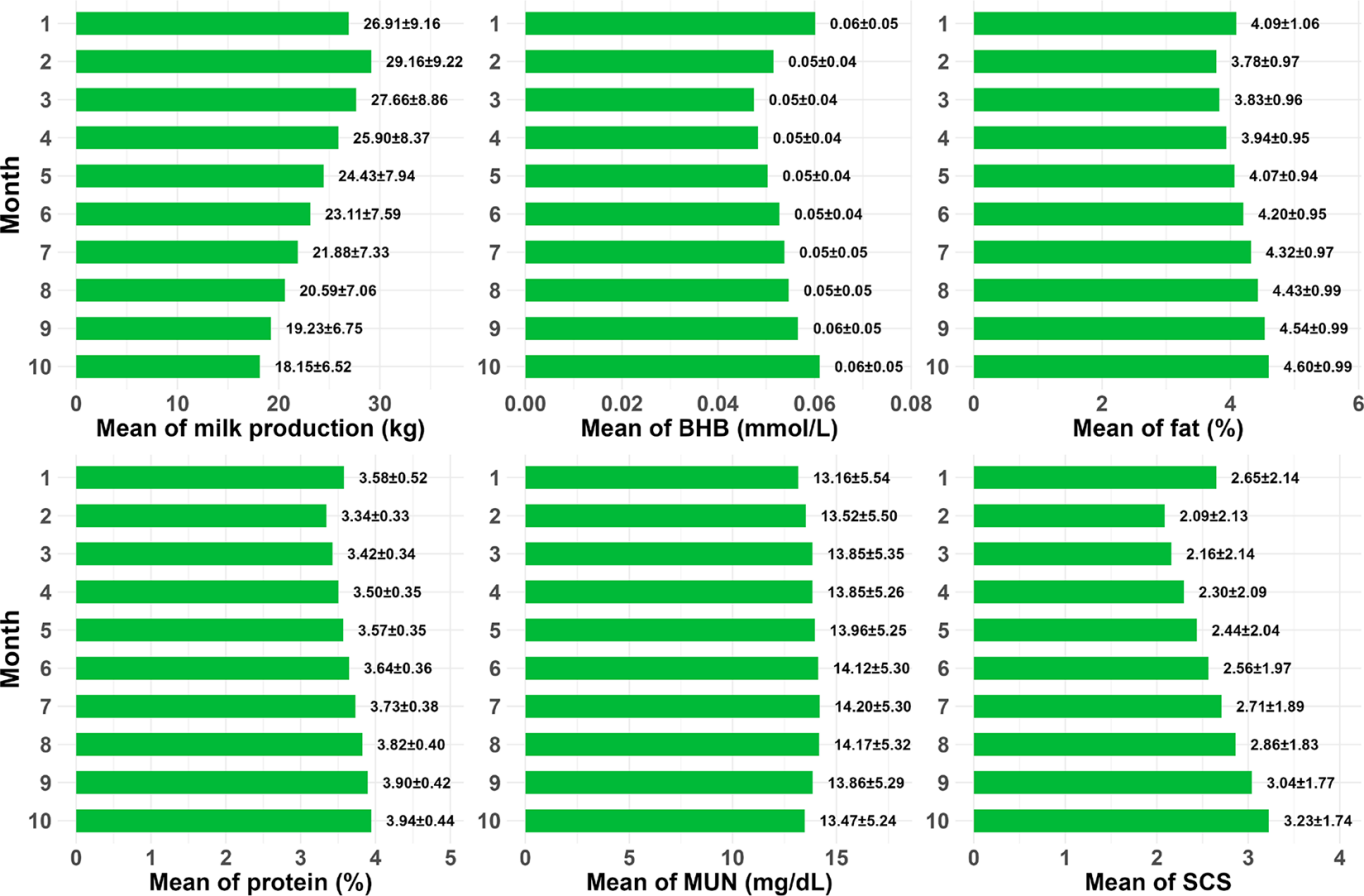
Phenotypic means of milk production and composition traits across the first 10 mo of lactation in the south region. The 6 panels correspond to milk production (kg), BHB (mmol/L), fat (%), protein (%), MUN (mg/dL), and SCS. All traits were measured from milk samples. Somatic cell score is a logarithmic transformation of the original SCC (1,000 cells/mL) based on the formula SCS = 3 + log_2_(SCC/100). The values depicted on each bar represent the mean ± SD for the month.

Our findings show that milk BHB concentrations in grazing cows (0.05–0.06 mmol/L) are substantially lower than those reported in confined dairy herds, representing a reduction of approximately ~22% to 66% compared with confined systems. For instance, a Canadian study encompassing 17 dairy herds observed a mean milk BHB concentration of 0.10 ± 0.06 mmol/L during the first 50 d postpartum. The enrolled cows produced an average of 36 ± 10 kg of milk per day, with 4.16% ± 1.38% fat, 3.33% ± 0.08% protein, and an SCC of 202,441 ± 518,193 cells/mL (Renaud et al., 2019). Similarly, Chin et al. (2024) reported a mean milk BHB concentration of 0.07 mmol/L in cows from 38 dairy herds in the Netherlands within the first 20 DIM. In a broader Canadian study involving 38,175 Holstein cows across 162 farms, the mean first-test milk BHB value was 0.11 ± 0.02 mmol/L (Van Soest et al., 2024). Additionally, De Jong et al. (2023) documented milk BHB concentrations ranging from 0.0003 to 1.19 mmol/L across 35 herds in the Netherlands, Belgium, and Luxembourg, with an average herd size of 89 cows (range: 14–290) and a mean daily milk yield of 29.7 kg per cow.

The heritability estimates for the 6 traits varied considerably across the lactation period (Figure 2). A shared pattern was observed for production traits, as the heritability for milk production, fat, and protein all followed an upward trend from early to mid or late lactation stage. The comparatively low heritability estimates for fat and protein percentage likely reflect the higher environmental variability characteristic of grazing-based systems, which inflates residual variance and reduces the proportion of phenotypic variance attributable to genetic effects. The estimate for milk production, for example, was initially 0.20 ± 0.007 at the early stage of lactation, which was consistent with previous reports (Belay et al., 2017; Benedet et al., 2020). It then steadily increased to a peak value above 0.40 at a middle stage of lactation. In contrast, the heritability of BHB followed a parabolic curve. At the earliest lactation stage, it had a low heritability of 0.12 ± 0.01 and showed an increasing trend up to mo 7, which was in line with previous reports (Koeck, et al., 2014). In contrast to the study of Koeck et al. (2016) that reported a heritability of 0.28 at 3 to 4 mo, our estimated BHB heritability peaked at 0.20 ± 0.007, suggesting a different pattern of genetic contribution of BHB for grazing cows across the lactation. Somatic cell score showed the lowest heritability in the early lactation stage at ~0.04 ± 0.003, but its genetic influence grew steadily throughout the period. Milk urea nitrogen also showed a relatively low heritability, which fluctuated during lactation.

**Figure 2:**
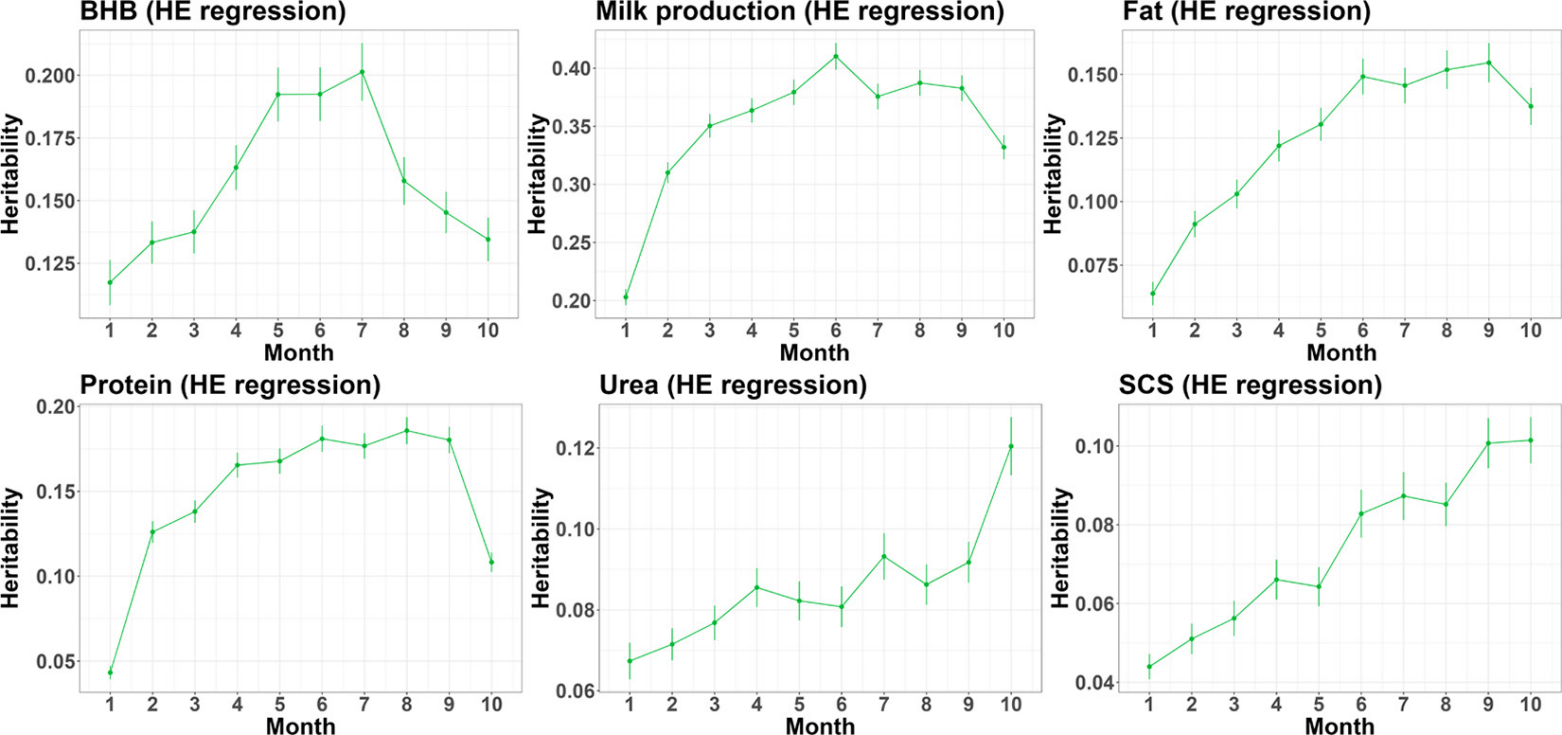
Monthly heritability estimates for milk production and composition traits across the first 10 mo of lactation in the south region. The 6 panels display heritability (h^2^) estimates for BHB, milk production, fat percentage, protein percentage, MUN, and SCS. All traits were derived from milk samples, with SCS being a logarithmic transformation of the original somatic cell count. Heritability was estimated using the Haseman–Elston (HE) regression method implemented in the GCTA tool. Vertical error bars represent the standard error of the heritability estimates.

Our study presents preliminary findings on the heritability of BHB concentrations in milk and their associations with additional traits observed in grazing dairy cattle. Although milk BHB does not perfectly mirror blood BHB concentrations, its measurement remains a cost-effective, rapid, and reliable method for assessing the energy status and fat-glucose metabolism of lactating cows. Indeed, studies have reported a strong correlation between milk and blood BHB levels, with coefficients of ~0.8 (de Roos et al., 2007; Renaud et al., 2019). This is particularly relevant in the context of genetic selection aimed at mitigating metabolic disorders such as ketosis. The heritability estimates observed in our study align with previous research, which reported values ranging from 0.07 to 0.19 (Koeck et al., 2016; Ranaraja et al., 2018). These varying heritability patterns suggest that the timing of genetic selection should be tailored to specific traits. To further investigate the consistency of milk BHB across lactation, we estimated its repeatability across test days. The repeatability estimate was low (0.173 ± 0.001), indicating that milk BHB concentrations are not stable within cows across lactation stages. This suggests that cows with elevated BHB in early lactation do not necessarily maintain higher values later in lactation. Therefore, BHB measurements in mid or late lactation are not reliable proxies for early lactation metabolic status and have limited value for selection against ketosis. Instead, early lactation remains the most biologically relevant period for evaluating metabolic disorders such as hyperketonemia. Nevertheless, although the heritability of BHB is lower during the first month of lactation compared with later months, this does not diminish the biological and clinical importance of elevated BHB concentrations during this period. Rather, the low heritability indicates that variation in milk BHB during early lactation is driven more by environmental than genetic factors. This can be advantageous, as appropriate management practices can effectively reduce the incidence of high BHB concentrations, thereby mitigating their adverse impact on production, health, and fertility. In contrast, the higher heritability observed in subsequent months is of limited relevance because by mid-lactation, cows typically reach a homeostatic equilibrium in which ketosis is no longer a major metabolic concern. In addition, it should be noted that very early lactation records (e.g., first 7–10 DIM) are often underrepresented in milk recording systems, which may limit the ability to fully capture peak BHB concentrations. These results highlight the distinction between the clinical relevance of milk BHB in early lactation and its genetic signal across lactation, which reflects changing contributions of genetic and environmental factors.

The genetic correlations between BHB and the other 5 traits revealed substantial and evolving relationships as lactation progressed (Figure 3). In most cases, the magnitude of the genetic correlations is greater than that of corresponding phenotypic correlations, indicating strong internal genetic links. The genetic correlation between BHB and milk production exhibited a clear reversal. It changed from a positive value of approximately 0.35 ± 0.02 in month 1 to a negative value of approximately −0.30 ± 0.03 by month 10, whereas the phenotypic correlation remained weakly negative throughout. A similar reversal was also observed for the genetic correlation between BHB and MUN. The genetic correlation between BHB and fat percentage was consistently positive through lactation, peaking above 0.30. For the health trait, the genetic correlation between BHB and SCS changed from near zero in the first few months to moderately positive in late lactation. This combination of opposite correlations reveals critical genetic antagonism. These results suggest that selection for higher milk yield may favor lower BHB levels in mid to late lactation; however, this relationship is stage-dependent and differs in early lactation, where the genetic correlation is positive. It should be acknowledged that fluctuations in genetic correlation estimates, particularly in later lactation, may partly reflect reduced genetic variance and smaller effective sample sizes, which can affect the stability of moment-based estimates. Nonetheless, standard errors remained small, and the overall temporal patterns were consistent across traits.

**Figure 3:**
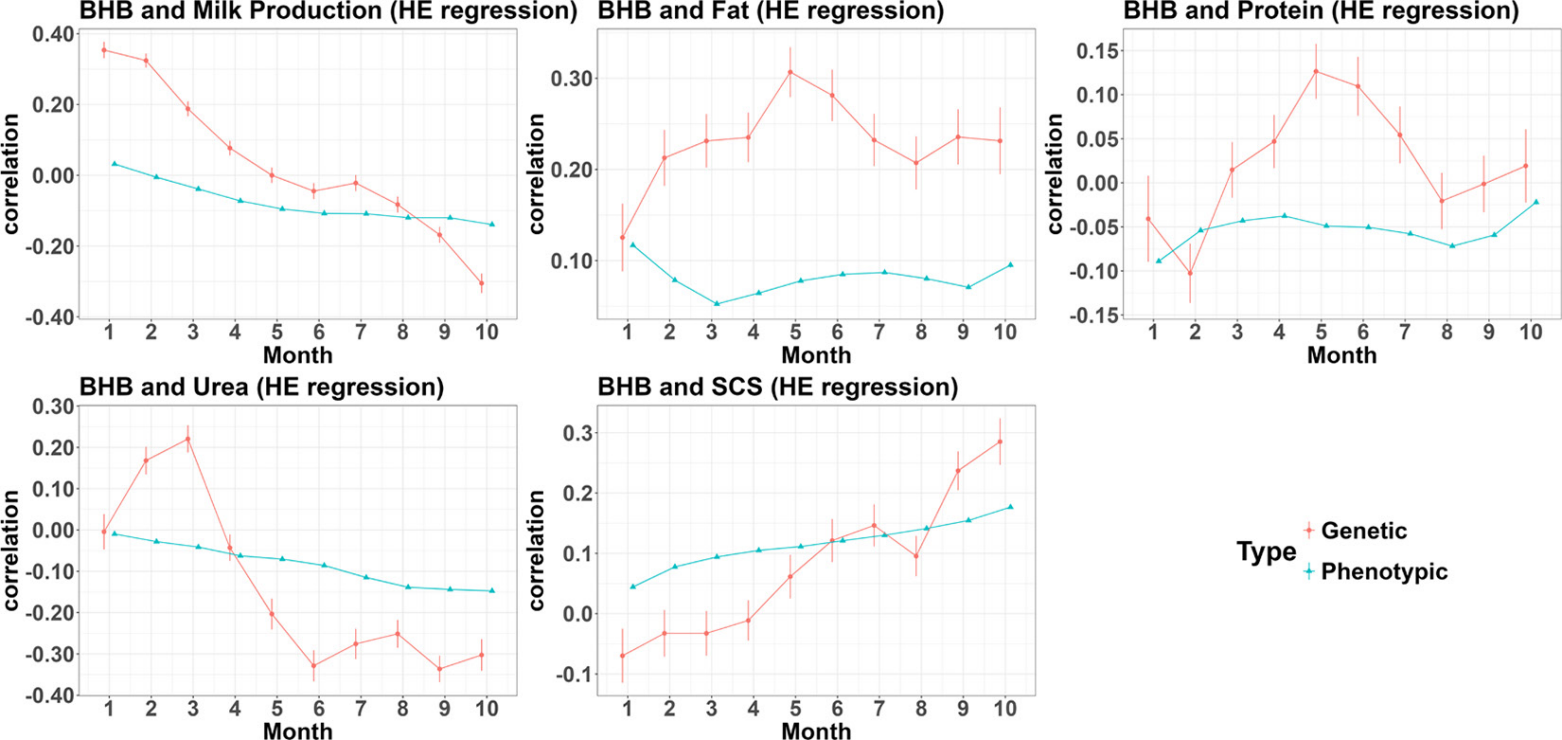
Genetic and phenotypic correlations between milk BHB and 5 key traits across the first 10 mo of lactation. The 5 panels display the monthly genetic (red line with circles) and phenotypic (blue line with triangles) correlations of BHB with milk production, fat percentage, protein percentage, MUN, and SCS. All traits were derived from milk samples. Correlation estimates were obtained via the Haseman–Elston (HE) regression method. Vertical error bars indicate the standard error of correlation estimates.
